# S1P induces proliferation of pulmonary artery smooth muscle cells by promoting YAP-induced Notch3 expression and activation

**DOI:** 10.1016/j.jbc.2021.100599

**Published:** 2021-03-26

**Authors:** Jian Wang, Xin Yan, Wei Feng, Qingting Wang, Wenhua Shi, Limin Chai, Qianqian Zhang, Yuqian Chen, Jin Liu, Zhan Qu, Xinming Xie, Manxiang Li

**Affiliations:** Department of Respiratory and Critical Care Medicine, First Affiliated Hospital of Xi’an Jiaotong University, Xi’an, Shaanxi, P.R. China

**Keywords:** sphingosine-1-phosphate, S1PR2, miR-135b, ubiquitination, YAP, Notch3, PASMCs, microRNA, STAT3, cell proliferation, β-TrCP, β-transduction repeat-containing protein, EdU, 5′-ethynyl-2′-deoxyuridine, NC, negative control, NICD3, intracellular domain of the Notch3, PAH, pulmonary arterial hypertension, PASMCs, pulmonary artery smooth muscle cells, qRT, quantitative real-time, S1P, sphingosine-1-phosphate, S1PR, S1P receptor, STAT3, signal transducers and activators of transcription 3, YAP, yes-associated protein

## Abstract

Sphingosine-1-phosphate (S1P), a natural multifunctional phospholipid, is highly increased in plasma from patients with pulmonary arterial hypertension and mediates proliferation of pulmonary artery smooth muscle cells (PASMCs) by activating the Notch3 signaling pathway. However, the mechanisms underpinning S1P-mediated induction of PASMCs proliferation remain unclear. In this study, using biochemical and molecular biology approaches, RNA interference and gene expression analyses, 5′-ethynyl-2′-deoxyuridine incorporation assay, and 3-(4,5-dimethylthiazol-2-yl)-2, 5-diphenyltetrazolium bromide assay, we demonstrated that S1P promoted the activation of signal transducers and activators of transcription 3 (STAT3) through sphingosine-1-phosphate receptor 2 (S1PR2), and subsequently upregulated the expression of the microRNA miR-135b, which further reduced the expression of E3 ubiquitin ligase β-transduction repeat-containing protein and led to a reduction in yes-associated protein (YAP) ubiquitinated degradation in PASMCs. YAP is the core effector of the Hippo pathway and mediates the expression of particular genes. The accumulation of YAP further increased the expression and activation of Notch3 and ultimately promoted the proliferation of PASMCs. In addition, we showed that preblocking S1PR2, prior silencing of STAT3, miR-135b, or YAP, and prior inhibition of Notch3 all attenuated S1P-induced PASMCs proliferation. Taken together, our study indicates that S1P stimulates PASMCs proliferation by activation of the S1PR2/STAT3/miR-135b/β-transduction repeat-containing protein/YAP/Notch3 pathway, and our data suggest that targeting this cascade might have potential value in ameliorating PASMCs hyperproliferation and benefit pulmonary arterial hypertension.

Pulmonary arterial hypertension (PAH) is a progressive disease characterized by histological changes of the pulmonary vasculature, such as sustained vasoconstriction, uncontrolled pulmonary vascular remodeling, and thrombosis *in situ*, resulting in structural and functional changes of the pulmonary vasculature, continuous and notable elevation of pulmonary vascular resistance and pulmonary arterial pressure, and eventually leads to heart failure and death (1, 2). Pulmonary artery smooth muscle cells (PASMCs) hyperproliferation is the most prominent feature of pulmonary vascular remodeling (3). Thus, understanding the precise mechanisms involved in PASMCs proliferation in pulmonary vascular remodeling is important for identifying novel therapeutics.

Sphingosine-1-phosphate (S1P) is a natural multifunctional phospholipid released by various cells, and sphingosine kinase 1 is a highly conserved lipid kinase for phosphorylating sphingosine to generate S1P (4). It has been found that the elevated level of S1P is consistent with the growth-promoting effects in many cancer cells (5, 6). Meanwhile, Gairhe *et al.* (7) have found that the plasma S1P level is increased in patients with idiopathic PAH and in early and late stages of PAH in rats. We have recently reported that sphingosine kinase 1/S1P mediates transforming growth factor-β1-induced PASMCs proliferation by activating the Notch3 signaling pathway (8). Notch3 is a transmembrane receptor; upon activation, the intracellular domain of the Notch3 (NICD3) receptor is released and translocated into the nucleus to regulate the transcription of specific target genes (9). Our previous studies have demonstrated that the Notch3 receptor is involved in the development of PAH by promoting the proliferation of PASMCs (8, 10, 11). However, the precise mechanism of how S1P activates the Notch3 signaling pathway remains unclear. Yes-associated protein (YAP) is the core protein of the Hippo signaling pathway and mediates various cell functions, including cell growth, survival, and differentiation (12). It has been reported that YAP is involved in the pathogenesis of PAH (13, 14). Meanwhile, studies have shown that YAP promotes differentiation of neural crest progenitors into smooth muscle cells during the development of the arterial wall by activating Notch signaling (15, 16). In addition, it has been shown that the contraction of muscle fibers increases the expression of YAP, which in turn activates Notch signaling in neighboring satellite cells, thereby preventing their differentiation (16, 17). S1P has been identified to activate YAP by inhibiting the Hippo pathway kinase Lats1/2 in HEK293A cells (18), and Liu *et al.* (19) have reported that S1P can induce the proliferation and migration of airway smooth muscle cells by activating YAP. Therefore, we hypothesized that S1P activates Notch3 signaling by upregulating YAP in PASMCs.

MicroRNAs are small noncoding RNAs that bind to the 3' untranslated regions of the target genes, thereby inhibiting translation and/or inducing degradation of target mRNAs. It has been shown that signal transducers and activators of transcription 3 (STAT3), an important downstream of S1P (20, 21), mediates the expression of miR-135b in lymphoma cells (22, 23). Meanwhile, miR-135b can downregulate the expression of the β-transduction repeat-containing protein (β-TrCP) in lung cancer cells (24) and hepatocellular carcinoma cells (25). The Skp1-Cullin-F-box ubiquitin ligase β-TrCP has been shown to be involved in many diseases including cancers and PAH (26, 27). In addition, studies have demonstrated that the ubiquitin ligase β-TrCP is mainly responsible for the ubiquitinated degradation and thus inhibit the function of YAP in mammalian cells (28, 29, 30). Therefore, we hypothesized that S1P upregulates YAP by activating STAT3/miR-135b/β-TrCP signaling pathway in PASMCs.

S1P regulates cellular activities *via* S1P receptors (S1PRs). Five types of S1PRs have been identified in mammalian cells, and S1PR2 and S1PR3 are the major types of S1PRs to transduce cellular effects of S1P in most of cell types (19, 31, 32, 33). To address above hypothesis and to clarify which S1PR is involved in this process, we intervened STAT3, miR-135b, β-TrCP, YAP, Notch3, and S1PR2/3 in primary cultured PASMCs stimulated with exogenous S1P, separately. We found that S1P increased the expression and activation of Notch3 by upregulating the protein level of YAP. Further experiments showed that the S1PR2/STAT3/miR-135b/β-TrCP signaling pathway mediated S1P induction of YAP protein and ultimately promoted PASMCs proliferation.

## Results

### Upregulation of YAP mediates S1P-induced Notch3 expression and activation

**Figure 1:**
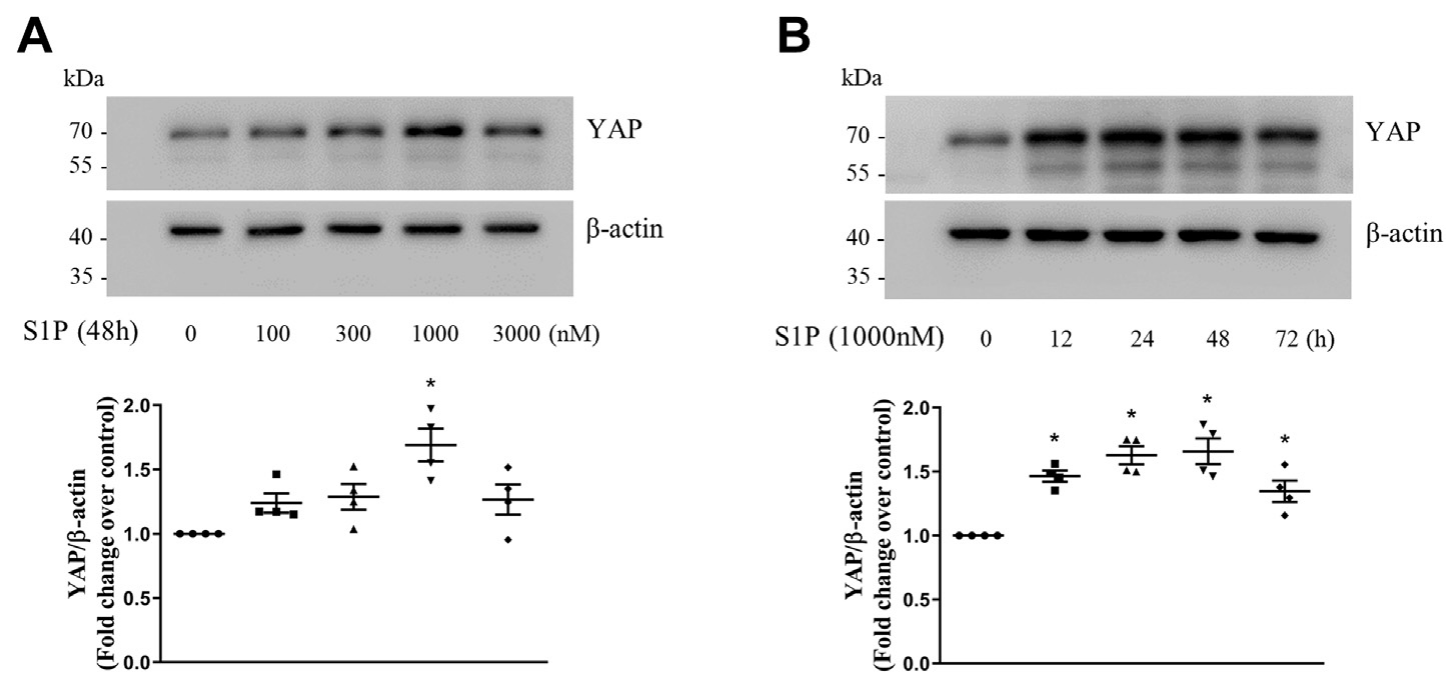
**S1P upregulates the protein level of YAP in PASMCs.***A*, PASMCs were stimulated with different concentrations of S1P in the range of 100 to 3000 nM for 48 h; the protein level of YAP was examined using Western blotting; β-actin served as a loading control (n = 4 each group). *B*, PASMCs were exposed to 1000-nM S1P for the indicated times; the protein level of YAP was detected using Western blotting; β-actin served as a loading control (n = 4 each group). ∗*p* < 0.05 *versus* control. PASMCs, pulmonary artery smooth muscle cells; S1P, sphingosine-1-phosphate; YAP, yes-associated protein.

To investigate whether S1P upregulates YAP expression in PASMCs, cells were treated with different concentration of S1P at different time points, and Western blotting was used to detect the protein level of YAP. As shown in Figure 1*A*, S1P increased the protein level of YAP in PASMCs at 48 h, and 1000-nM S1P triggered a 1.69-fold increase compared with the control. Figure 1*B* shows that 1000-nM S1P significantly increased YAP protein levels from 12 h to 48 h, which reached to 1.46-fold compared with the control at the time of 12 h, 1.63-fold at 24 h, and 1.66-fold at 48 h. These results suggest that S1P upregulates the protein level of YAP in PASMCs.

**Figure 2:**
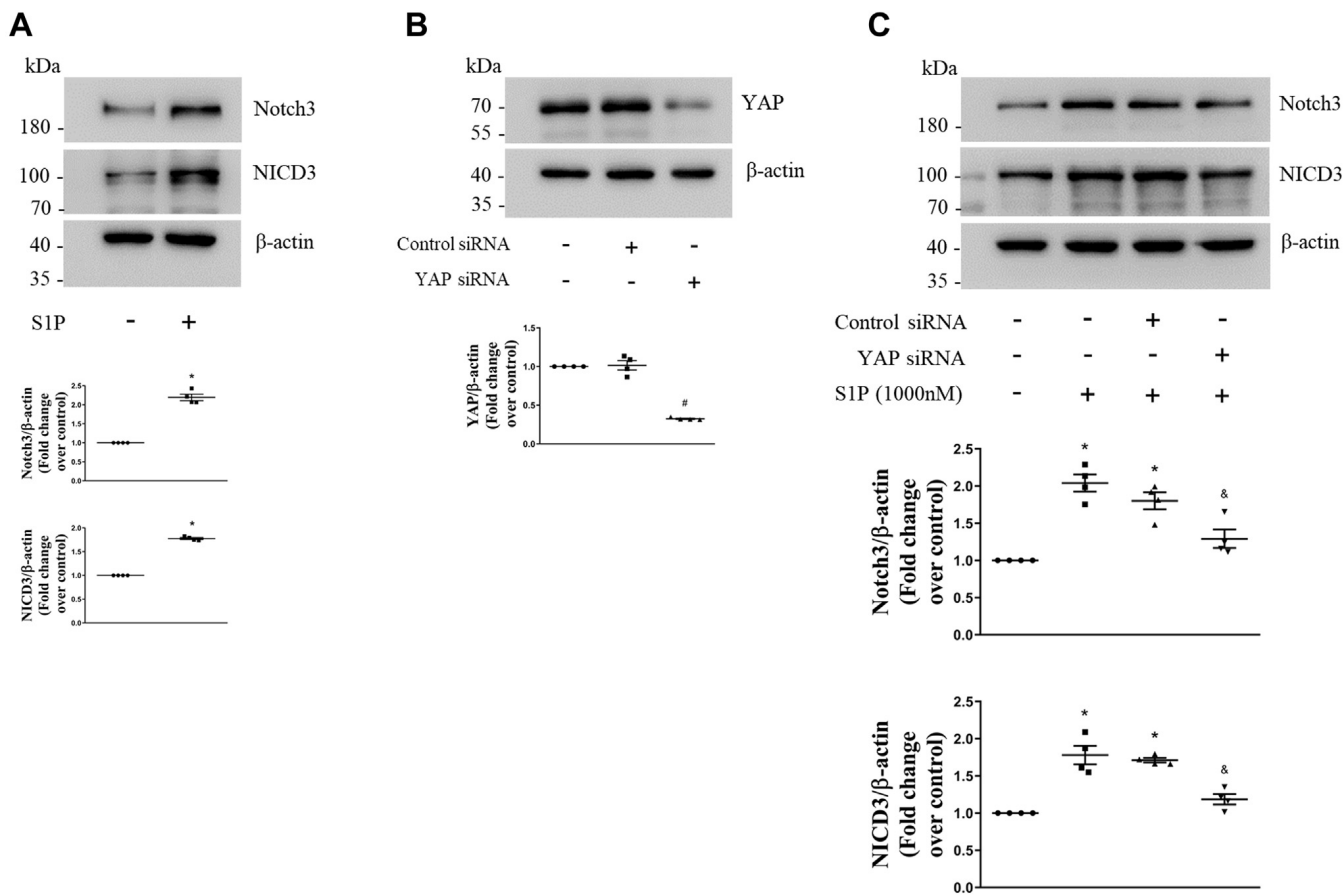
**YAP mediates S1P-induced Notch3 expression and activation in PASMCs.***A*, PASMCs were treated with 1000-nM S1P for 48 h; the protein levels of Notch3 and NICD3 were examined using Western blotting; β-actin served as a loading control (n = 4 each group). *B*, PASMCs were transfected with YAP sequence-specific siRNA or nontargeting siRNA; the YAP protein level was determined using Western blotting; β-actin served as a loading control (n = 4 each group). *C*, PASMCs were previously transfected with YAP-specific or nontargeting siRNA for 24 h and then stimulated with 1000-nM S1P for 48 h; protein levels of Notch3 and NICD3 were detected using Western blotting; β-actin served as a loading control (n = 4 each group). ∗*p* < 0.05 *versus* control, #*p* < 0.05 *versus* control siRNA, &*p* < 0.05 *versus* control siRNA and S1P-cotreated cells. NICD3, intracellular domain of the Notch3; PASMCs, pulmonary artery smooth muscle cells; S1P, sphingosine-1-phosphate; YAP, yes-associated protein.

We next investigated whether S1P promotes Notch3 expression and activation in PASMCs. First, cells were stimulated with 1000-nM S1P for 48 h, and protein levels of Notch3 and the NICD3 were determined using Western blotting. As shown in Figure 2*A*, 1000-nM S1P stimulation for 48 h resulted in a 2.19-fold increase in the Notch3 protein level compared with the control, and a 1.78-fold increase in the NICD3 protein level. To further clarify whether the upregulation of YAP mediates the effect of S1P on the expression and activation of Notch3 in PASMCs, siRNA-mediated YAP knockdown was performed and protein levels of YAP, Notch3, and the NICD3 were examined. Figure 2*B* shows that transfection of YAP siRNA for 48 h in PASMCs reduced the YAP protein level to 32%, whereas transfection of negative control (NC) siRNA did not change the YAP protein level. Figure 2*C* indicates that 1000-nM S1P stimulation for 48 h significantly increased Notch3 and the NICD3 protein levels, whereas prior knockdown of YAP notably reduced S1P-induced increases of Notch3 and the NICD3 protein levels in PASMCs, which decreased from 1.80-fold and 1.71-fold in control siRNA and S1P-cotreated cells to 1.29-fold and 1.19-fold in YAP siRNA and S1P-cotreated cells, separately. Together, these results suggest that the upregulation of YAP mediates S1P-induced Notch3 expression and activation in PASMCs.

### STAT3 mediates S1P-induced increase of YAP and the expression and activation of Notch3

**Figure 3:**
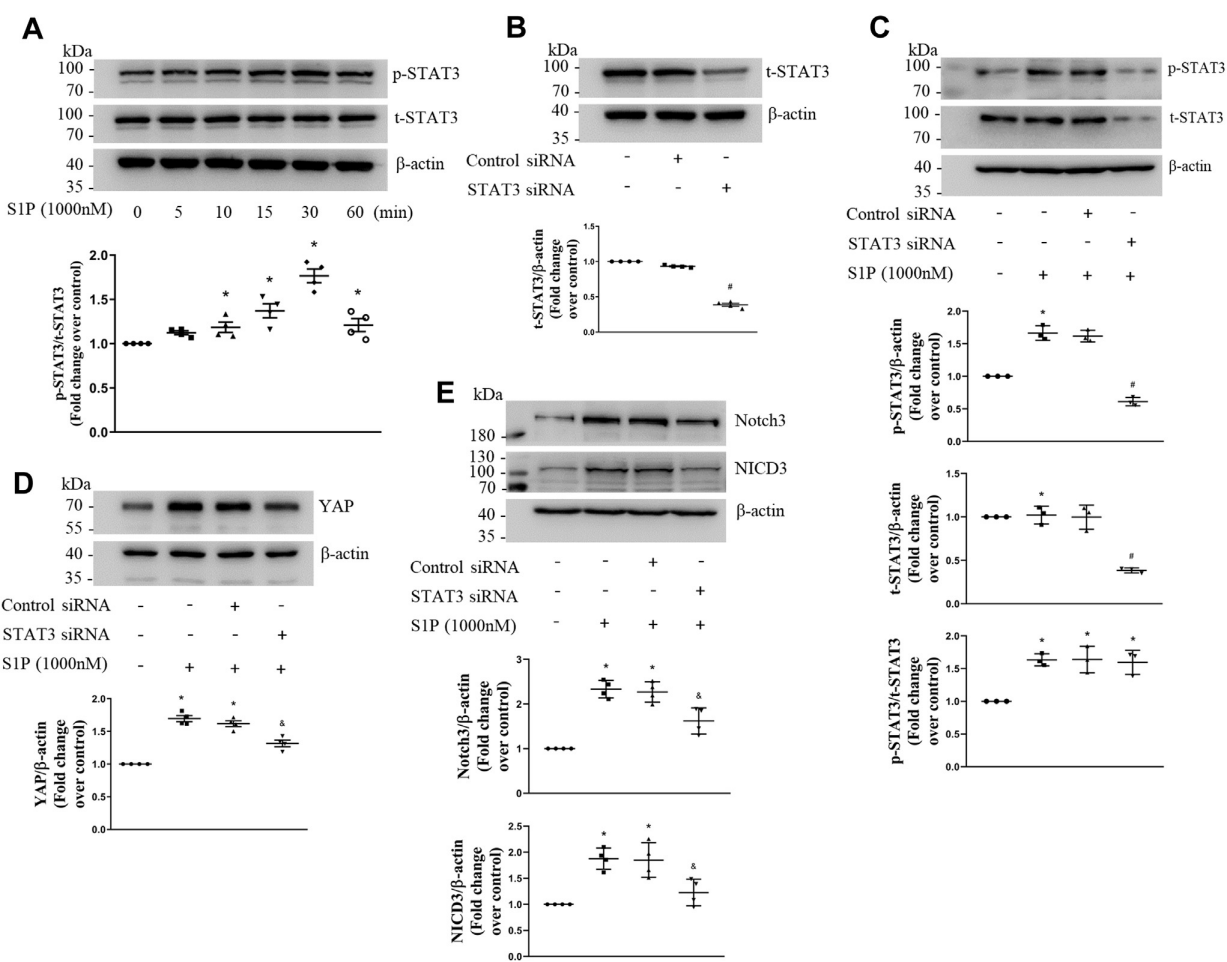
**STAT3 mediates S1P-induced increase of YAP and Notch3 expression and activation.***A*, PASMCs were exposed to 1000-nM S1P for the indicated times; the phosphorylation level of STAT3 and total STAT3 protein level were examined using Western blotting; β-actin served as a loading control (n = 4 each group). *B*, PASMCs were transfected with STAT3 sequence-specific siRNA or nontargeting siRNA; the total STAT3 protein level was determined using Western blotting; β-actin served as a loading control (n = 4 each group). *C*, PASMCs were previously transfected with STAT3-specific or nontargeting siRNA for 48 h and then stimulated with 1000-nM S1P for 30 min; the phosphorylation of STAT3 and total STAT3 were determined using Western blotting; β-actin served as a loading control (n = 3 each group). *D* and *E*, PASMCs were previously transfected with STAT3-specific or nontargeting siRNA for 24 h and then treated with 1000-nM S1P for 48 h; protein levels of YAP, Notch3, and NICD3 were examined using Western blotting; β-actin served as a loading control (n = 4 each group). ∗*p* < 0.05 *versus* control, #*p* < 0.05 *versus* control siRNA, &*p* < 0.05 *versus* control siRNA and S1P-cotreated cells. NICD3, intracellular domain of the Notch3; PASMCs, pulmonary artery smooth muscle cells; S1P, sphingosine-1-phosphate; STAT3, signal transducers and activators of transcription 3; YAP, yes-associated protein.

Studies have shown that S1P promotes the activation of STAT3 in non-PASMCs (20, 21). To examine the effect of S1P on STAT3 in PASMCs, cells were stimulated with 1000-nM S1P at different time points, and Western blotting was performed to detect the phosphorylation level of STAT3 and total STAT3 level. Figure 3*A* shows that S1P significantly increased the phosphorylation of STAT3 from 10 min to 60 min, which reached to 1.77-fold compared with the control at 30 min. This suggests that S1P activates STAT3 in PASMCs.

To further investigate whether STAT3 mediates S1P-induced increase of YAP and the expression and activation of Notch3, siRNA-mediated STAT3 knockdown was performed and phosphorylation and protein level of STAT3, protein levels of YAP, Notch3, and NICD3 were detected using Western blotting. As shown in Figure 3*B*, transfection of STAT3 siRNA for 48 h in PASMCs reduced the STAT3 protein level to 39%, whereas transfection of NC siRNA did not affect the STAT3 protein level. Figure 3*C* shows that preknockdown of STAT3 with siRNA for 48 h notably reduced S1P-induced increase of phosphorylation of STAT3 in PASMCs, which reduced from 1.62-fold over control to 0.61-fold over control. This suggests that phosphorylated STAT3 is sensitive to STAT3 siRNA knockdown. Figure 3*D* indicates that 1000-nM S1P stimulation for 48 h significantly increased the protein level of YAP, whereas prior silencing of STAT3 notably reduced the elevated protein level of YAP induced by S1P in PASMCs, which dropped from 1.62-fold over control in control siRNA and S1P-cotreated cells to 1.32-fold over control in STAT3 siRNA and S1P-cotreated cells. And Figure 3*E* shows that loss of STAT3 notably reduced S1P-induced Notch3 and NICD3 protein levels in PASMCs, which decreased from 2.27-fold and 1.85-fold in control siRNA and S1P-cotreated cells to 1.62-fold and 1.23-fold in STAT3 siRNA and S1P-cotreated cells, separately. Taken together, the above results indicate that STAT3 mediates S1P-induced increase of YAP protein and the expression and activation of Notch3.

### STAT3 mediates S1P-induced miR-135b upregulation

**Figure 4:**
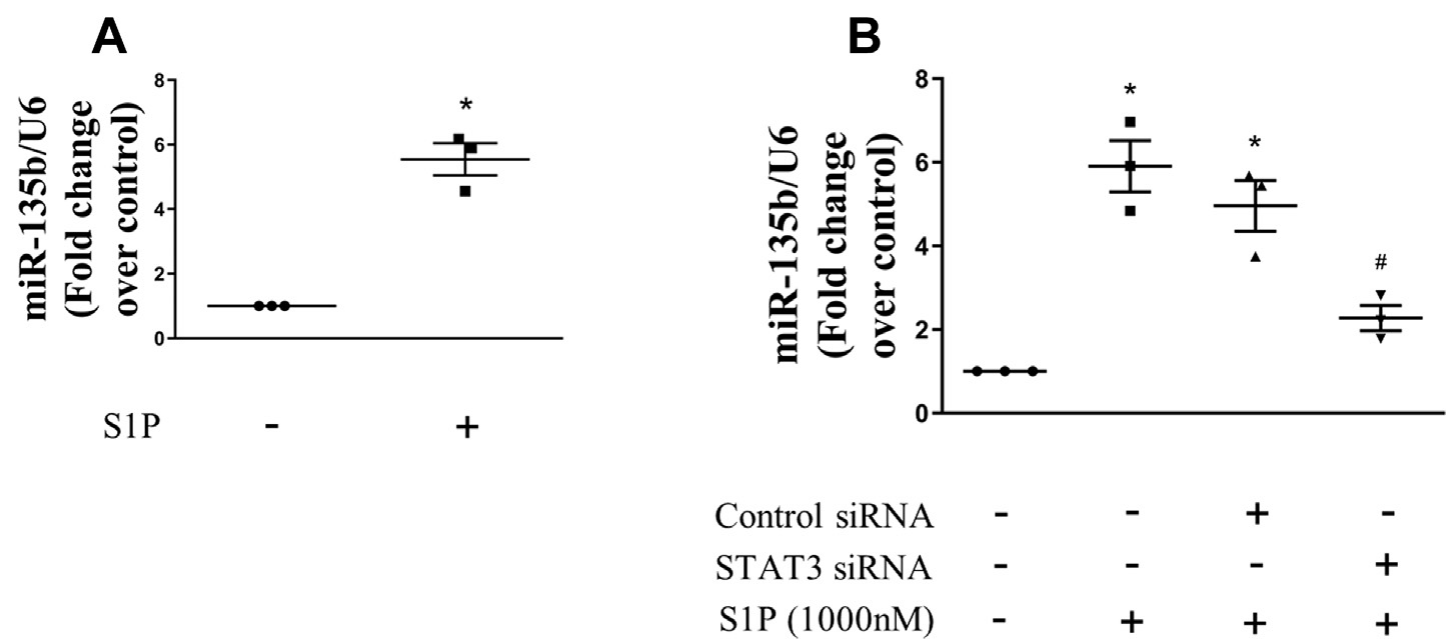
**STAT3 mediates S1P-induced miR-135b upregulation.***A*, PASMCs were stimulated with 1000-nM S1P for 24 h; the expression of miRNA-135b was examined using qRT-PCR, and U6 small nuclear RNA served as a loading control (n = 3 each group). *B*, PASMCs were previously transfected with STAT3-specific or nontargeting siRNA for 24 h and then treated with 1000-nM S1P for 24 h; the expression of miRNA-135b was determined using qRT-PCR, and U6 served as a loading control (n = 3 each group). ∗*p* < 0.05 *versus* control, #*p* < 0.05 *versus* control siRNA and S1P-cotreated cells. PASMCs, pulmonary artery smooth muscle cells; qRT, quantitative real-time; S1P, sphingosine-1-phosphate; STAT3, signal transducers and activators of transcription 3.

Studies have reported that STAT3 mediates the expression of miR-135b in lymphoma cells (22). To examine whether S1P promotes miR-135b upregulation in PASMCs, the level of miR-135b was examined using quantitative real-time (qRT)-PCR. As shown in Figure 4*A*, 1000-nM S1P stimulation for 24 h resulted in a 5.54-fold increase in miR-135b expression compared with the control. To further verify whether STAT3 mediates this effect, siRNA-mediated STAT3 knockdown was performed, and the level of miR-135b was determined. Figure 4*B* indicates that prior knockdown of STAT3 dramatically reduced the elevated expression of miR-135b induced by S1P from 4.96-fold in control siRNA and S1P-cotreated cells to 2.27-fold in STAT3 siRNA and S1P-cotreated cells. These results suggest that STAT3 mediates S1P-induced miR-135b upregulation.

### S1P suppresses β-TrCP expression by upregulating miR-135b

**Figure 5:**
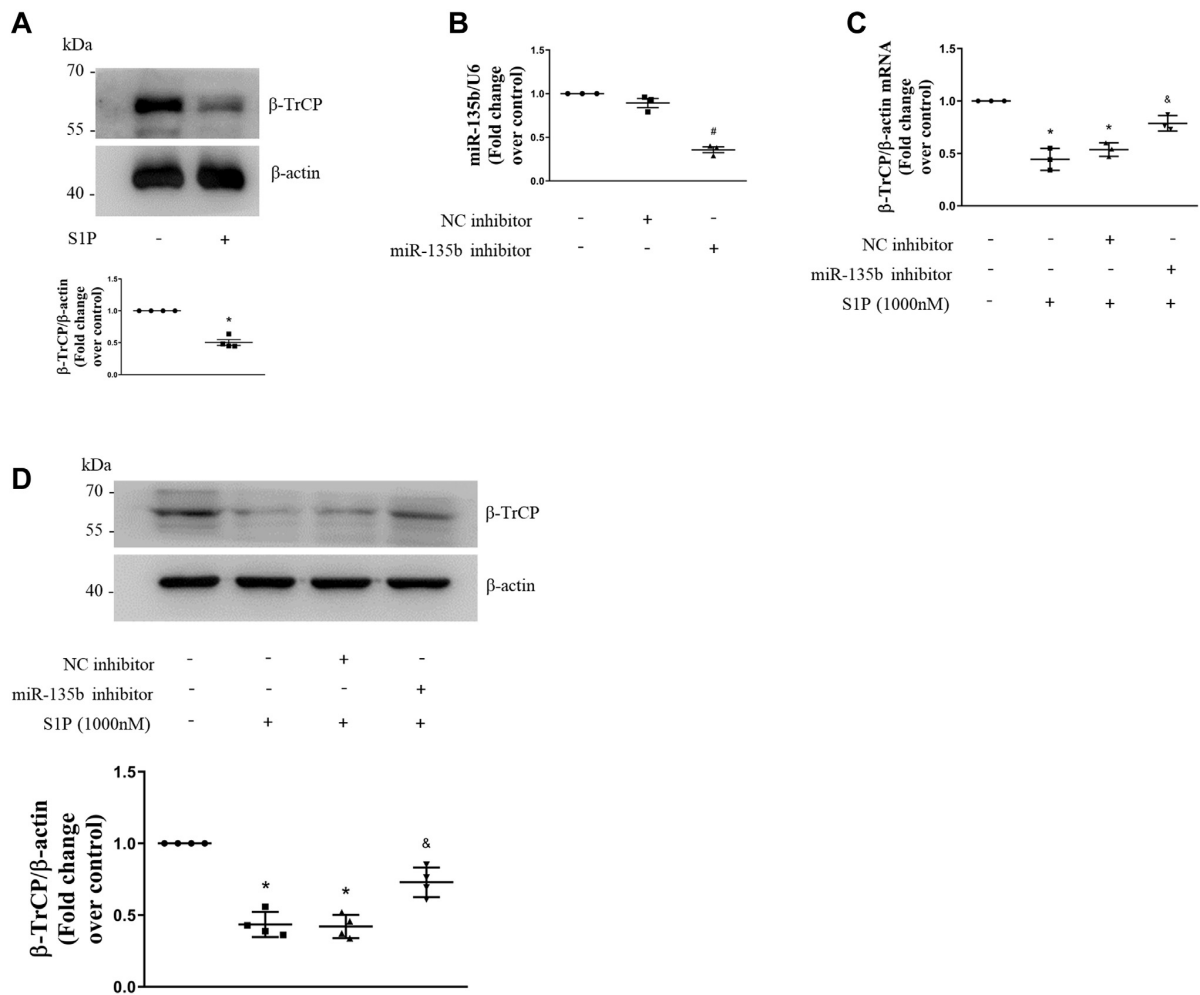
**S1P suppresses β-TrCP expression by upregulating miR-135b.***A*, PASMCs were stimulated with 1000-nM S1P for 48 h; the protein level of β-TrCP was examined using Western blotting, β-actin served as a loading control (n = 4 each group). *B*, PASMCs were transfected with the miRNA-135b inhibitor or negative control inhibitor; the expression of miRNA-135b was detected using qRT-PCR, U6 served as a loading control (n = 3 each group). *C*, PASMCs were previously transfected with the miRNA-135b inhibitor or negative control inhibitor for 24 h and then treated with 1000-nM S1P for 48 h; mRNA level of β-TrCP was detected by qRT-PCR, and β-actin served as a control (n = 3 each group). *D*, PASMCs were previously transfected with miRNA-135b inhibitor or negative control inhibitor for 24 h and then treated with 1000-nM S1P for 48 h; the protein level of β-TrCP was detected using Western blotting, β-actin served as a loading control (n = 4 each group). ∗*p* < 0.05 *versus* control, #*p* < 0.05 *versus* negative control inhibitor, &*p* < 0.05 *versus* negative control inhibitor and S1P-cotreated cells. β-TrCP, β-transduction repeat-containing protein; PASMCs, pulmonary artery smooth muscle cells; qRT, quantitative real-time; S1P, sphingosine-1-phosphate.

It has been reported that platelet-derived growth factor, a key mediator in the pathogenesis of PAH, downregulates the expression of the β-TrCP in PASMCs (26). To investigate whether S1P also inhibits β-TrCP expression in PASMCs, the cells were stimulated with 1000-nM S1P for 48 h and the protein level of the β-TrCP was examined using Western blotting. As shown in Figure 5*A*, S1P reduced the protein level of β-TrCP to 0.50-fold over control. This suggests that S1P inhibits β-TrCP expression in PASMCs.

Studies have shown that miR-135b targets and downregulates the expression of the β-TrCP in non-PASMCs (24, 25). To clarify whether S1P-induced miR-135b upregulation mediates the reduction of the β-TrCP in PASMCs, sequence-specific inhibitor-mediated miR-135b knockdown was performed and the levels of miR-135b and β-TrCP mRNA were determined using qRT-PCR, and the protein level of the β-TrCP was examined using Western blotting. As shown in Figure 5*B*, transfection with the miR-135b sequence-specific inhibitor for 48 h reduced the miR-135b level to 36% of the control in PASMCs, whereas NC miRNA inhibitor transfection did not change the miR-135b level. Figure 5, *C* and *D* shows that 1000-nM S1P stimulation for 48 h significantly decreased the mRNA level and protein level of the β-TrCP, whereas prior knockdown of miR-135b reversed S1P-induced reduction of the mRNA level and protein level of the β-TrCP, which increased from 0.54-fold and 0.42-fold in the NC inhibitor and S1P-cotreated cells to 0.79-fold and 0.73-fold in miR-135b inhibitor and S1P-cotreated cells, separately. These results indicate that S1P suppresses β-TrCP expression by upregulating miR-135b.

### β-TrCP promotes the ubiquitinated degradation of YAP in PASMCs

**Figure 6:**
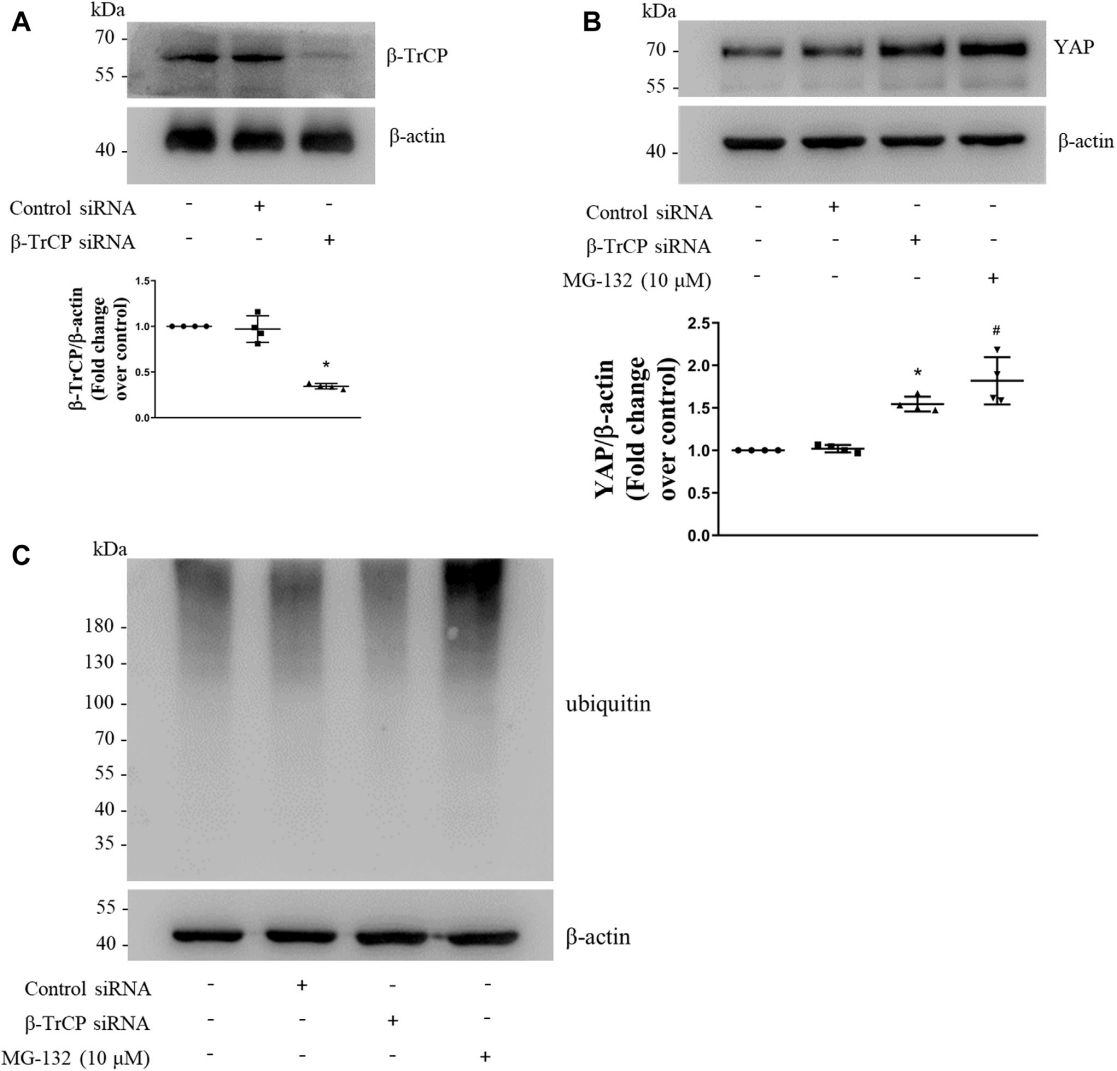
**β-TrCP promotes the ubiquitinated degradation of YAP in PASMCs.***A*, PASMCs were transfected with β-TrCP sequence-specific siRNA or nontargeting siRNA; β-TrCP protein level was examined using Western blotting; β-actin served as a loading control (n = 4 each group). *B*, PASMCs were transfected with β-TrCP sequence-specific siRNA or nontargeting siRNA or proteasome inhibitor MG-132; protein level of YAP was determined using Western blotting; β-actin served as a loading control (n = 4 each group). *C*, PASMCs were transfected with β-TrCP sequence-specific siRNA or nontargeting siRNA or proteasome inhibitor MG-132; ubiquitinated proteins were detected using Western blotting; β-actin served as a loading control (n = 4 each group). ∗*p* < 0.05 *versus* control siRNA, #*p* < 0.05 *versus* control. β-TrCP, β-transduction repeat-containing protein; PASMCs, pulmonary artery smooth muscle cells; YAP, yes-associated protein.

It has been shown that the β-TrCP mediates the ubiquitinated degradation of YAP and thus inhibits the function of YAP in non-PASMCs (28, 29, 30). To investigate whether the β-TrCP promotes the ubiquitinated degradation of YAP in PASMCs, sequence-specific siRNA was used to silence the β-TrCP, and proteasome inhibitor MG-132 was used to inhibit ubiquitin proteasome (34), and protein levels of the β-TrCP and YAP were examined using Western blotting. As shown in Figure 6*A*, transfection of β-TrCP siRNA for 48 h in PASMCs reduced the β-TrCP level to 34%, whereas transfection of NC siRNA did not change β-TrCP level. Figure 6*B* shows that prior silencing of the β-TrCP significantly increased the YAP protein level to 1.54-fold compared with the control and inhibiting the ubiquitin proteasome system with 10-μM MG-132 for 12 h also increased the YAP protein level to 1.81-fold over control. Figure 6*C* shows that the loss of β-TrCP reduced ubiquitinated proteins including YAP, whereas inhibition of ubiquitin proteasome increased the accumulation of ubiquitinated YAP and other proteins. The results of our study suggest that the β-TrCP promotes the ubiquitinated degradation of YAP, and it may be inferred that the reduction of the β-TrCP induced by S1P mediates the elevated protein level of YAP in PASMCs.

### STAT3/miR-135b/β-TrCP/YAP/Notch3 mediates S1P-induced proliferation of PASMCs

**Figure 7:**
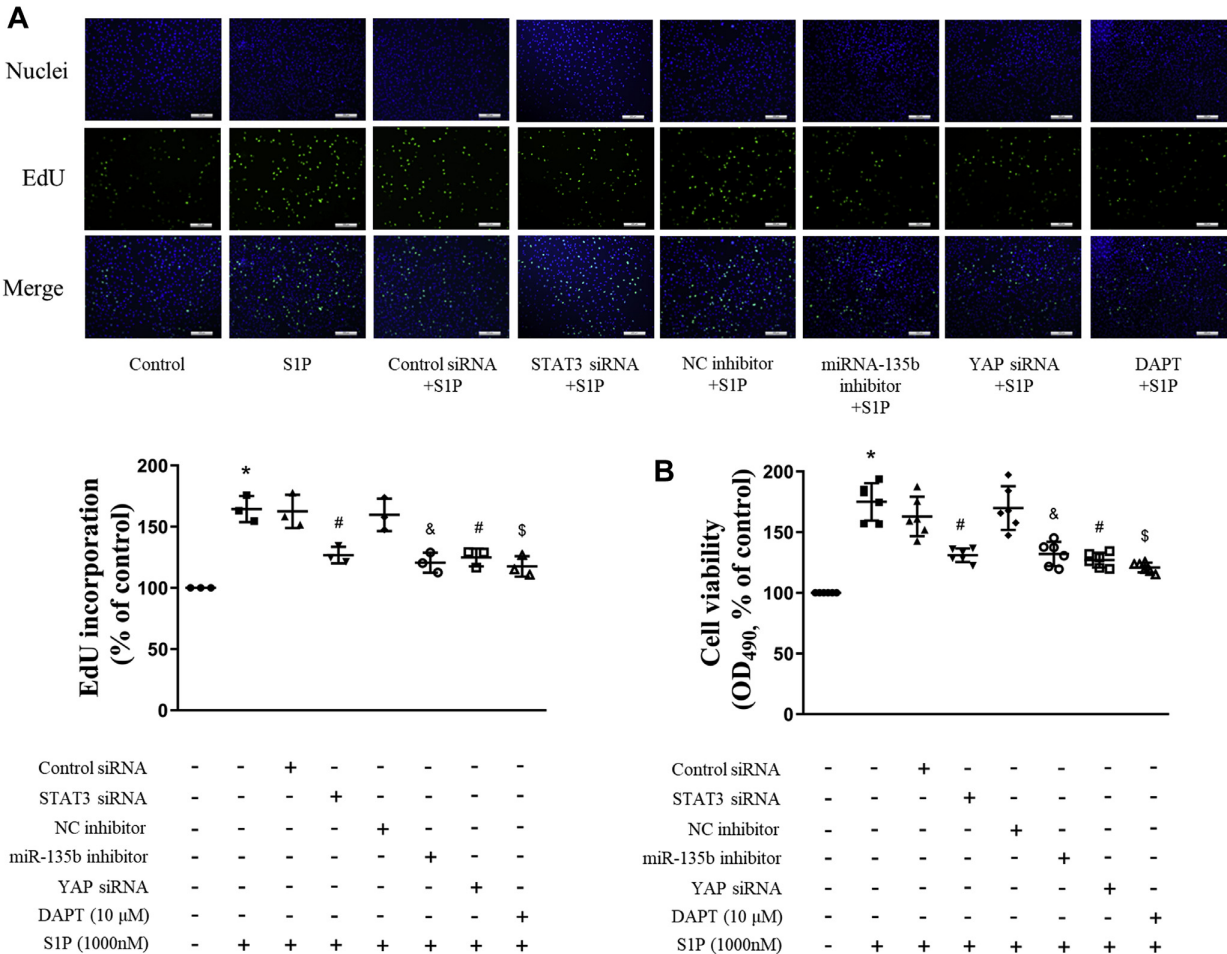
**STAT3/miR-135b/β-TrCP/YAP/Notch3 mediates S1P-induced proliferation of PASMCs.** PASMCs were previously transfected with STAT3 siRNA or miR-135b inhibitor or YAP siRNA for 24 h or preincubated with 10-μM DAPT for 4 h and then stimulated with 1000-nM S1P for an additional 48 h, the proliferation of cells was evaluated by EdU incorporation assay (*A*, the scale bar represents 200 μm, n = 3 each group) and cell viability assay (*B*, n = 6 each group). ∗*p* < 0.05 *versus* control, #*p* < 0.05 *versus* control siRNA and S1P-cotreated cells, &*p* < 0.05 *versus* negative control inhibitor and S1P-cotreated cells, $*p* < 0.05 *versus* S1P-treated cells. β-TrCP, β-transduction repeat-containing protein; DAPT, N-[N-(3, 5-difluorophenacetyl)-l-alanyl]-s-phenylglycinet-butyl ester; EdU, 5′-ethynyl-2′-deoxyuridine; PASMCs, pulmonary artery smooth muscle cells; STAT3, signal transducers and activators of transcription 3; YAP, yes-associated protein.

To clarify whether activation of STAT3, upregulation of miR-135b, accumulation of YAP induced by the reduction of the β-TrCP, and subsequent Notch3 expression and activation are involved in S1P-induced PASMCs proliferation, cells were previously transfected with STAT3 siRNA or the miR-135b inhibitor or YAP siRNA for 24 h, or preincubated with 10-μM N-[N-(3, 5-difluorophenacetyl)-l-alanyl]-s-phenylglycinet-butyl ester for 4 h, and then stimulated with 1000-nM S1P for an additional 48 h; the proliferation of cells were evaluated by 5′-ethynyl-2′-deoxyuridine (EdU) incorporation assay and cell viability assay. As shown in Figure 7*A*, interfering the STAT3/miR-135b/β-TrCP/YAP/Notch3 axis significantly suppressed PASMCs proliferation assessed by EdU staining, and the percentage of EdU-positive cells was reduced from 1.65-fold over control in S1P-treated cells to 1.27-fold, 1.21-fold, 1.25-fold, and 1.18-fold over control, separately. Similar results were observed by the PASMCs viability assay (Fig. 7*B*), which was reduced from 1.75-fold in S1P-treated cells to 1.31-fold, 1.32-fold, 1.27-fold, and 1.21-fold over control, separately. These results suggest that STAT3/miR-135b/β-TrCP/YAP/Notch3 cascade mediates S1P-induced PASMCs proliferation.

### S1PR2 mediates S1P-induced changes of the STAT3/miR-135b/β-TrCP/YAP/Notch3 signal pathway and PASMCs proliferation

**Figure 8:**
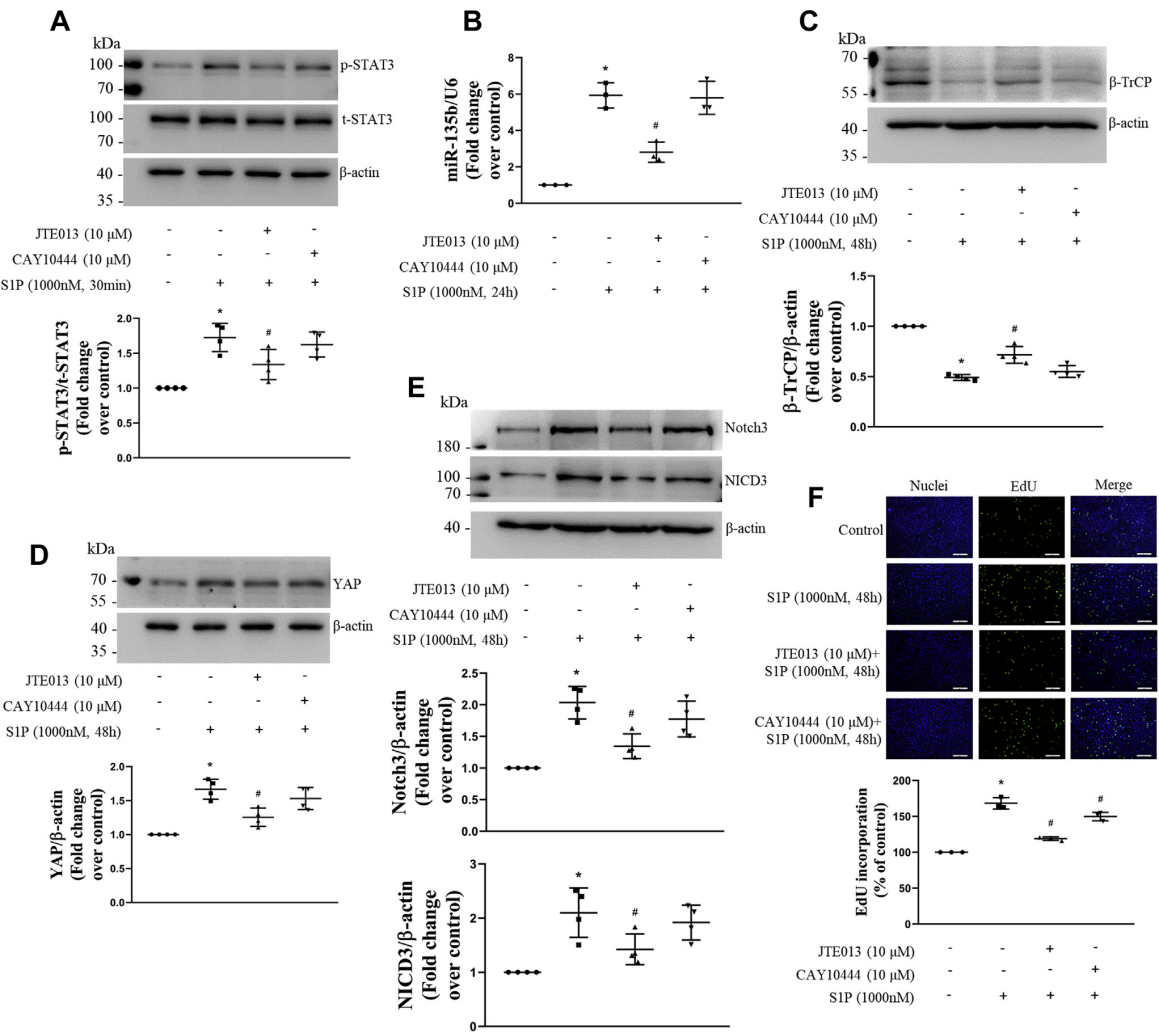
**S1PR2 mediates S1P-induced changes of the STAT3/miR-135b/β-TrCP/YAP/Notch3 signal pathway and PASMCs proliferation.** PASMCs were previously treated with JTE013 (10-μM) or CAY10444 (10-μM) for 1 h and then stimulated with S1P, the phosphorylation level of STAT3 (*A*) and total STAT3 protein level (*A*), protein levels of β-TrCP (*C*), YAP (*D*), Notch3 (*E*) and NICD3 (*E*) were examined using Western blotting; β-actin served as a loading control (n = 4 each group); mRNA level of miRNA-135b (*B*) was detected using qRT-PCR; U6 served as a loading control (n = 3 each group); proliferation of PASMCs (*F*) was evaluated by the EdU incorporation assay (the scale bar represents 200 μm, n = 3 each group). ∗*p* < 0.05 *versus* control, # *p* < 0.05 *versus* S1P-treated cells. β-TrCP, β-transduction repeat-containing protein; EdU, 5′-ethynyl-2′-deoxyuridine; NICD3, intracellular domain of the Notch3; PASMCs, pulmonary artery smooth muscle cells; qRT, quantitative real-time; S1P, sphingosine-1-phosphate; STAT3, signal transducers and activators of transcription 3; YAP, yes-associated protein.

Studies have demonstrated that S1PR2 and S1PR3 are the major types of S1PRs to transduce cellular effects of S1P in most of mammalian cell types (19, 31, 32, 33). To examine whether S1PR2 and S1PR3 mediate S1P-induced activation of the STAT3/miR-135b/β-TrCP/YAP/Notch3 signal pathway and PASMCs proliferation, cells were previously treated with JTE013 (a selective S1PR2 antagonist, 10-μM) or CAY10444 (a selective S1PR3 antagonist, 10-μM) for 1 h and then stimulated with S1P, the phosphorylation level and total protein level of STAT3 and protein levels of β-TrCP, YAP, Notch3, and NICD3 were examined using Western blotting, the level of miRNA-135b was detected using qRT-PCR, and proliferation of cells was evaluated by the EdU incorporation assay. As shown in Figure 8, inhibition of S1PR2 significantly reduced S1P-induced increase of STAT3 phosphorylation from 1.73-fold to 1.34-fold over control; decreased the S1P-caused miRNA-135b elevation from 5.93-fold to 2.81-fold over control; increased the S1P-induced downregulation of β-TrCP from 0.49-fold to 0.72-fold over control; and reduced S1P-resulted increase of YAP, Notch3, and NICD3 from 1.67-fold, 2.03-fold, and 2.10-fold to 1.25-fold, 1.34-fold, and 1.42-fold over control, separately. However, prior blocking of S1PR3 did not affect these changes induced by S1P. In addition, preblocking S1PR2 reduced S1P-induced PASMCs proliferation from 1.64-fold to 1.20-fold over control. Interestingly, presuppression of S1PR3 also slightly but statistically inhibited S1P-triggered PASMCs proliferation to 1.43-fold over control. Taken together, these results suggest that S1PR2 mediates S1P-induced activation of the STAT3/miR-135b/β-TrCP/YAP/Notch3 signal pathway and proliferation of PASMCs, and S1PR3 may partial mediate S1P-induced PASMCs proliferation by other signal pathways.

## Discussion

S1P is a natural multifunctional phospholipid secreted by a variety of cells that regulates various cellular processes including cell proliferation, differentiation, apoptosis, migration, invasion, and angiogenesis (5, 35). It has been found that elevation of S1P is associated with the growth-promoting effects in many cancer cells (5, 6). Meanwhile, Gairhe *et al.* (7) have found that the plasma S1P level is increased in patients with idiopathic PAH and in early and late stages of PAH in rats. We have recently reported that the elevated level of S1P mediates transforming growth factor-β1-induced proliferation of PASMCs by activation of the Notch3 signaling pathway (8). Notch3 and NICD3 have been shown to participate in the development of PAH by promoting PASMCs proliferation and pulmonary vascular remolding (10, 11). The present study confirmed that S1P significantly promoted proliferation of PASMCs by increasing Notch3 expression and activation. This is consistent with the results showed in other cell types (36, 37). Hirata *et al.* (37) have reported that S1P promotes expansion of cancer stem cells by activating the Notch signaling pathway. Our study verifies the importance of S1P in the pathophysiological process of PAH.

YAP is a core protein of the Hippo signaling pathway and is involved in the pathogenesis of many diseases (13, 19, 38). With the YAP elevation and activation, more YAP protein translocates into the nucleus and promotes the expression of particular target genes (39). High level of YAP has been detected in ovarian, colon, gastric, liver, esophageal, and non–small-cell lung cancers and lobular type of invasive breast cancers (38). Meanwhile, it has been shown that the expression of YAP protein in primary cultured PASMCs from patients with idiopathic PAH was significantly raised (13). In addition, S1P has been identified to activate YAP by inhibiting the Hippo pathway kinase Lats1/2 in HEK293A cells (18). In this study, we demonstrated that S1P significantly upregulated the protein level of YAP in PASMCs, and YAP further mediated S1P-induced Notch3 expression and activation and PASMCs proliferation. These results are consistent with published studies; Manderfield *et al.* (15) have shown that YAP promotes differentiation of neural crest progenitors into smooth muscle cells during the development of the arterial wall by activating Notch signaling; Esteves de Lima *et al.* (17) have proved that the enhancement of YAP expression can activate Notch signaling in neighboring satellite cells, thereby preventing their differentiation. Our results indicate that YAP is required in S1P-triggered PASMCs hyperproliferation by upregulation and activation of Notch3 signaling.

STAT3 is a transcription factor involved in many signaling pathways of different cell types under a wide variety of conditions (40, 41). STAT3 is mainly activated by phosphorylation of the conserved Tyr705 residue, and activated STAT3 further translocates to the nucleus to regulate the expression of multiple genes (40, 42), such as *Hif1α*, *LEMD1*, and *miR-135b* (22, 43). S1P has been found to play an essential role in maintaining persistent activation of STAT3 in cancer cells (44, 45). In this study, we demonstrated that S1P significantly promoted the phosphorylation of STAT3 in PASMCs, which further mediated S1P-induced upregulation of miR-135b and YAP, and the expression and activation of Notch3. Upregulation of miR-135b has been detected in a variety of cancers including breast cancer, non–small-cell lung cancer, prostate cancer, and colon cancer (23, 24, 46, 47, 48). It has been reported that upregulation of miR-135b is far more robust in highly invasive non–small-cell lung cancer cell lines than the less-invasive cell lines, and a high level of miR-135b in lung cancer specimens is significantly correlated with decreased overall survival (24). Meanwhile, Lin *et al.* (24) have shown that miR-135b promotes the progression of non–small-cell lung cancer by targeting multiple key components in the Hippo pathway, including LZTS1, MOB1, and β-TrCP. Here, we demonstrated that the STAT3/miR-135b pathway mediated S1P-induced PASMCs hyperproliferation.

Recently, we have shown that the reduction of β-TrCP mediates PDGF-induced PASMCs hyperproliferation by increasing the expression of cell division cycle 25 homologue A (26). As a Skp1-Cullin-F-box ubiquitin ligase substrate recognition subunit, β-TrCP is responsible for the ubiquitination of several substrate proteins which are then targeted for proteasomal degradation (27, 49). The β-TrCP exerts pivotal roles in regulating various key cellular processes including cell proliferation, apoptosis, and differentiation (49). Many of the signaling steps are coordinated by protein ubiquitination, including the Hippo/YAP signal pathway (28). When the Hippo pathway is on, activated LATS1/2 phosphorylates YAP, and then β-TrCP ligase is recruited, leading to YAP ubiquitination and degradation (28, 29). In this study, we showed that S1P reduced the expression of β-TrCP by STAT3/miR-135b pathway and increased YAP expression, and knockdown of β-TrCP increased the YAP protein level and reduced ubiquitinated proteins including YAP, while inhibition of ubiquitin proteasome increased the accumulation of ubiquitinated YAP and other proteins. Together, these results indicate that β-TrCP mediated YAP ubiquitinated degradation in PASMCs, and it may be inferred that the downregulation of β-TrCP induced by S1P was responsible for elevated YAP and PASMCs hyperproliferation.

S1P regulates cellular activities *via* five types of S1PRs in mammalian cells, and S1PR2 and S1PR3 are the major types to transduce cellular effects of S1P in most of cell types (19, 31, 32, 33). Chen *et al.* (33) have reported that S1P promotes human PASMCs proliferation *via* S1PR2, and blocking S1PR2 with JTE013 prevents the development of PAH in hypoxia mice. In this study, we demonstrated that S1PR2 was the predominant receptor that mediated S1P-induced activation of the STAT3/miR-135b/β-TrCP/YAP/Notch3 signal pathway and proliferation of PASMCs.

**Figure 9:**
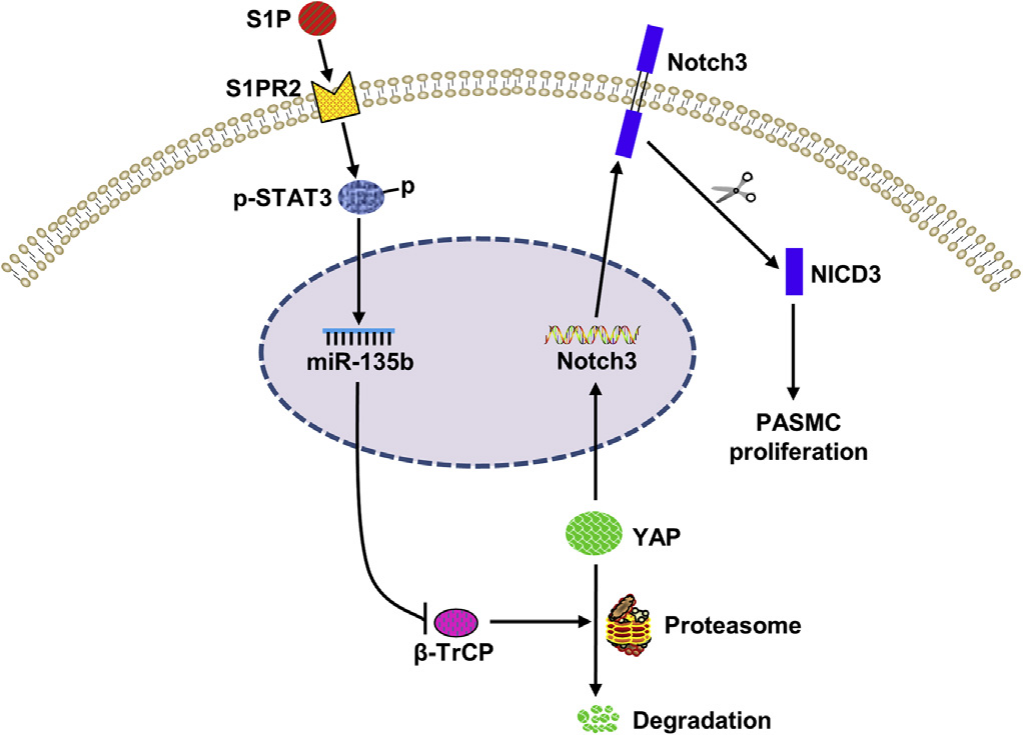
**The molecular mechanisms of S1P-induced rat PASMCs proliferation.** S1P promotes the activation of STAT3 through S1PR2 and subsequently upregulates the expression of miR-135b, which further reduces the expression of β-TrCP and leads to a reduction in YAP ubiquitinated degradation. The accumulation of YAP further increases the expression and activation of Notch3 and ultimately promotes the proliferation of PASMCs. β-TrCP, β-transduction repeat-containing protein; NICD3, intracellular domain of the Notch3; PASMCs, pulmonary artery smooth muscle cells; S1P, sphingosine-1-phosphate; STAT3, signal transducers and activators of transcription 3; YAP, yes-associated protein.

In conclusion, as outlined in Figure 9, we have demonstrated that S1P significantly induced PASMCs proliferation by activating the S1PR2/STAT3/miR-135b/β-TrCP/YAP/Notch3 pathway. Our study suggests that targeting S1P/S1PR2/STAT3/miR-135b/β-TrCP/YAP/Notch3 might have potential value in ameliorating PASMCs hyperproliferation and benefit PAH.

## Experimental procedures

### Cell preparation and culture

Primary cultured PASMCs were obtained from Sprague-Dawley male rats that weighed from 70 to 80 g by the method reported previously (8, 50). All animal care and experiments were performed in accordance with the *Guide for the Care and Use of Laboratory Animals* of Xi’an Jiaotong University Animal Experiment Center. All protocols used in this research were approved by the Laboratory Animal Care Committee of Xi’an Jiaotong University. Briefly, pulmonary arteries were rapidly isolated from killed rats and washed in PBS (4 °C). The adventitia was carefully stripped off with fine forceps, and the endothelium was carefully removed by scratching the intima surface with elbow tweezers. The remaining smooth muscle layer was cut into 1 mm^3^ pieces and placed into a culture flask with Dulbecco’s modified Eagle medium (DMEM; Gibco) with 10% fetal bovine serum (Gemini Bio-Products), 100 U/ml penicillin, and 100 μg/ml streptomycin and incubated at 37 °C in a humidified 5% CO_2_ incubator until the cells reached 80% confluence. Then, cells were passaged using 0.25% trypsin (Beyotime Biotechnology). Cells between passages two and six were used for further experiments. The purity of rat PASMCs was determined by immunostaining with α-smooth muscle actin (Boster Biological technology) as described previously (8, 51). Fluorescence microscope images showed that the cells contained more than 90% of PASMCs. Before each experiment, cells were serum-starved (1% fetal bovine serum in DMEM) overnight to minimize serum-induced effects. S1P (Cayman Chemical) was used to stimulate PASMCs. N-[N-(3, 5-difluorophenacetyl)-l-alanyl]-s-phenylglycinet-butyl ester (Santa Cruz Biotechnology), a γ-secretase inhibitor, was used to block the Notch pathway (8, 11). JTE013 (a selective S1PR2 antagonist) (31) and CAY10444 (a selective S1PR3 antagonist) (19) were purchased from Cayman Chemical.

### Oligonucleotide synthesis and transfection

The STAT3 siRNA, miR-135b inhibitor, β-TrCP siRNA, YAP siRNA, and NC oligonucleotides were synthesized by GenePharma. The sequences are as follows: STAT3 siRNA, sense 5′-CCCGCCAACAAAUUAAGAATT-3′, anti-sense 5′-UUCUUAAUUUGUUGGCGGGTT-3′; miR-135b inhibitor, 5′-UCACAUAGGAAUGAAAAGCCAUA-3′; β-TrCP siRNA, sense 5′-GCACAUCAACUCCUAUCUATT-3′, anti-sense 5′-UAGAUAGGAGUUGAUGUGCTT-3′; YAP siRNA, sense 5′-CUGCCACCAAGCUAGAUAATT-3′, anti-sense 5′-UUAUCUAGCUUGGUGGCAGTT-3′; NC siRNA, sense 5′-UUCUCCGAACGUGUCACGUTT-3′, anti-sense 5′-ACGUGACACGUUCGGAGAATT-3′; miRNA inhibitor NC, 5′-CAGUACUUUUGUGUAGUACAA-3′. Lipofectamine 2000 reagent (Invitrogen) was used for transfection according to the manufacturer’s protocols. Western blotting was used to analyze the effect of siRNA transfection, and qRT-PCR was used to analyze the effect of miRNA inhibitor transfection.

### Western blotting

Cells were washed twice with ice-cold PBS and then lysed in RIPA lysis buffer (Hat Biotechnology). Lysates were centrifuged at 15,000 rpm at 4 °C for 15 min, and the supernatants were collected as sample proteins. Proteins were separated on 8 to 12% SDS-PAGE gel and transferred onto polyvinylidene fluoride membranes (Millipore). After blocking for 1 h at room temperature (RT) with 5% nonfat milk, the membranes were incubated overnight at 4 °C with primary antibodies against p-STAT3 (Tyr705, 1:800 dilution; Cell Signaling Technology), t-STAT3 (1:1000 dilution; Cell Signaling Technology), β-TrCP (1:500 dilution; ABclonal), YAP (1:1000 dilution; Proteintech), Notch3 and NICD3 (1:500 dilution; Abcam), and β-actin (1:1000 dilution; Santa Cruz Biotechnology). Horseradish peroxidase–conjugated goat anti-rabbit (1:5000 dilution; Sigma Aldrich) or goat anti-mouse (1:5000 dilution; Santa Cruz Biotechnology) was used as the secondary antibody. Immunoreactive bands were visualized using the enhanced chemiluminescence kit (Millipore). Images were digitally captured using a ChemiDoc XRS System (Bio-Rad). The band densities were quantified using Quantity One software (Bio-Rad).

### qRT-PCR

Total RNA including the miRNA fraction was extracted from PASMCs using TRIzol Reagent (Invitrogen) following the manufacturer’s instructions. Isolated RNA was polyadenylated using the PrimeScript RT reagent Kit (Perfect Real Time; TaKaRa). The synthesized cDNA was used to perform qRT-PCR on StepOnePlus Real-Time PCR System (Thermo Fisher Scientific) using TB Green Premix Ex Taq II (Tli RNaseH Plus, TaKaRa). Primers specific for miR-135b, U6 small nuclear RNA, β-TrCP, and β-actin were purchased from Dingguo Biotechnology; the following primer sets were used: miR-135b, RT primer: 5′-GTCGTATCCAGTGCGTGTCGTGGAGTCGGCAATTGCACTGGATACGACTCACATA-3′, forward: 5′-GCGTATGGCTTTTCATTCCTA-3′, reverse: 5′-CAGTGCGTGTCGTGGAGT-3′; U6, RT primer: 5′-CCTGCTTCGGCAGCACAT-3′, forward: 5′-CCTGCTTCGGCAGCACAT-3′, reverse: 5′-AAATATGGAACGCTTCACG-3′; β-TrCP, forward: 5′-TGCCCAAGCAGCGGAAAC-3′, reverse: 5′-CAGCATAGGCTTTAGATAGGAG-3′; β-actin, forward: 5′-TTACTGCCCTGGCTCCTAG-3′, reverse: 5′-CGTACTCCTGCTTGCTGATC-3′. The fold increase relative to the control samples was determined by the 2^−ΔΔCt^ method. U6 served as an internal control for miR-135b; β-actin was used as an internal control for β-TrCP. Amplification was performed at 95 °C for 30 s, followed by 40 cycles of 95 °C for 5 s, 60 °C for 30 s, and 72 °C for 30 s.

### EdU incorporation assay

To determine PASMCs proliferation, the rate of EdU incorporation was examined using the EdU Cell Proliferation Kit (Beyotime Biotechnology) following the established protocol. PASMCs were planted into 24-well plates with a density of 2.5 × 10^4^ cells/well and allowing to adhere for at least 24 h. After serum starved overnight and different treatments, EdU was added to the wells and incubated for 2 h at 37 °C. Cells were then washed with 3% BSA three times and fixed with 4% paraformaldehyde for 10 min. After washing with 3% BSA three times again, cells were permeabilized with 0.3% Triton X-100 for 15 min at RT. Cells were then incubated with EdU staining cocktail (Click Reaction Buffer, CuSO_4_, Azide 488, Click Additive Solution) kept from lights at RT for 30 min. After washing with 3% BSA three times, samples were then counterstained with Hoechst 33342 and kept from lights at RT for 10 min. Images were acquired by using a fluorescence microscope. Cells stained both green (EdU-positive) and blue (nuclei) were considered proliferated cells.

### Cell viability

Cell viability was measured using 3-(4,5-dimethylthiazol-2-yl)-2, 5-diphenyltetrazolium bromide assay. Briefly, PASMCs were planted into 96-well plates at 5 × 10^3^ cells/well and allowed to adhere for at least 24 h. After serum starved overnight and different treatments, 20-μl 3-(4,5-dimethylthiazol-2-yl)-2, 5-diphenyltetrazolium bromide (5 mg/ml, PBS as a vehicle; GenView) was added into each well and incubated with cells at 37 °C for another 4 h. Then, 150-μl dimethyl sulfoxide was added to dissolve the formazan crystals. Finally, the microplate reader (Thermo) was used to measure the absorbance at 490 nm to quantify the reaction product.

### Statistical analysis

Statistical analysis was performed using the SPSS 22.0 software. All data were shown as the mean ± SD and analyzed using one-way ANOVA with Tukey's post hoc test. *p* < 0.05 was considered to represent a significant difference between groups.

## Data availability

All data are contained within the article.

## Conflict of interest

The authors declare that they have no conflicts of interest with the contents of this article.
